# Characteristics and Expression Profiles of Identified *WRKY* Genes in Barley Landraces Under Cold Stress

**DOI:** 10.3390/ijms26146948

**Published:** 2025-07-19

**Authors:** Yuancheng Zhou, Yiling Wang, Ting Gao, Yongli Cao, Yong Yao, Yukun Zhao, Zhen Wang

**Affiliations:** 1 Institute of Wheat Research, Shanxi Agricultural University, Linfen 041000, China; zhouyuancheng@sxau.edu.cn (Y.Z.); wangzhen3@sxau.edu.cn (Z.W.); 2 School of Life Science, Shanxi Normal University, Taiyuan 030031, China

**Keywords:** *WRKY*, barley landrace, cold stress, response

## Abstract

The *WRKY* gene family comprises important transcription factors widely distributed in plants and plays significant roles in the growth and development, diverse (biotic and abiotic) stress responses, and various biological processes. In the current study, 96 identified *HvLWRKY* genes were classified into three groups and seven subgroups. Among these, 89 genes possessed the conserved domain WRKYGQK. A total of ten motifs were harbored in *HvLWRKY* genes with two to four introns. Fragmental duplication was suggested to be the prime force that drove the evolution of *HvLWRKY* genes. A high degree of collinearity was observed between barley and *Triticum spelta*. *Cis*-elements of HvLWRKYs were closely associated with abiotic stress, light response, and hormone response; however, there were differences in the numbers among groups. *HvLWRKY* genes, even the paralogous gene pairs, from different clades were differentially regulated under cold treatments in two landraces. *HvLWRKY33*, *43*, *44*, *57*, *65*, and *77* were homologous with the relative *AtWRKY* genes in *Arabidopsis thaliana*. They are suggested to regulate abiotic and pathogen resistance of two barley landraces via SA and JA pathways. Meanwhile, some genes (for example, *HvLWRKY1* and *HvLWRKY32*) were specifically expressed in either cold-tolerant or cold-sensitive landraces. Under cold stress, different cold-responsive patterns occurred in different barley landraces. These findings provide a foundation for further studies on cold resistance in barley landraces and offer new insights for application of *WRKY* genes in barley breeding.

## 1. Introduction

Plants often encounter various biotic and abiotic stresses, including extreme temperatures, drought, and salinity [1,2,3,4]. These stresses severely prevent the plants from optimal growth and development, which have a great impact on yield production in crops [5,6,7]. To cope with such stresses, plants have developed diverse adaptive response mechanisms [8,9]. One of the mechanisms is through the intricate signaling system to rapidly respond to external factors. During the process, the key signaling system molecules are transcription factors (TFs) [9,10,11]. After receiving signals, stress-related TFs are activated to regulate the target genes’ expression through binding to the specific *cis*-elements in the gene promoter regions, and then participate in the response to abiotic stress [12,13,14,15,16]. To date, diverse TF families, including *WRKY*, C2H2, MYB and TCP, have been identified and functionally characterized [17,18,19,20].

Among the TF families, one of the largest families is *WRKY* TFs [21,22,23], which were first identified in plants [2,19,20,24,25]. The initial *WRKY* transcription factor, SPF1, was discovered in sweet potatoes (*Ipomoea batatas*) [24]. Subsequently, *WRKY1*, *WRKY2*, and *WRKY3* were identified in parsley (*Petroselinum crispum*) [26]. Recently, a large number of *WRKY* genes have been identified in more than 150 plant species [27]. Moreover, the number of *WRKY* genes significantly varies among plants. The lowest number of *WRKY* genes (<20) has been found in algae and some lower plant species [27]. With the availability of more genomic data, the number of *WRKY* genes has increased. *Glycine max* (296) possesses the highest quantity of *WRKY* genes, followed by *Brassica napus* (285) and *Panicum virgatum* (275) [28,29].

*WRKY* genes are widely implicated in the regulation of plant growth and development, including flowering, pollens, senescence, stems, and seed development and germination [19,30,31,32,33,34]. For example, in *WRKY75*, a positive regulator of leaf senescence, its function loss can delay leaf senescence [18], whereas *AtWRKY70* from *Arabidopsis thaliana* acts as a negative regulator of developmental senescence [35]. Meanwhile, many *WRKY* genes also participate in various stress responses [36,37,38,39,40]. Among thirteen *OsWRKY* genes of rice regulated by salt, polyethylene glycol (PEG), and cold or heat stresses, ten *WRKY* genes have been downregulated or upregulated by those abiotic stresses [41]. In *Vitis vinifera* L., the *VvWRKY11* gene can respond to dehydration stress, and the *VvWRKY24* gene can be induced by cold stress [42]. Under salt stress, the expression of *TaWRKY75-A* was highly induced in wheat (*Triticum aestivum*) [43,44]. Thus, the significant role of WRKYs in regulating abiotic stress and plant species’ growth and development has made them a popular gene family for plant stress breeding research. Recently, numerous studies have been conducted to uncover the impact of various stresses on WRKYs in diverse plants [2,9,45,46,47,48,49,50].

Barley (*Hordeum vulgare* L.) is the fourth most important cereal crop according to its yield production and cultivated area [7,51]. Two-thirds of its production was used for animal feed, with the remaining allocated to the malting and brewing industries, as well as the food industry in the Himalayas and some African countries, owing to the abundant dietary fiber and functional food constituents [52,53]. Like most crops, the growth, development and yield of barley are impaired by adverse circumstances and factors, such as, extreme temperatures, salinity and water stress [6,7]. Low temperature is recognized as a major environmental stress [11,25]. Cold stress, including chilling (<20 °C) and freezing (<0 °C), significantly limits barley productivity, particularly due to late spring frosts [54]. Different barley landraces can adapt to marginal conditions and provide a reservoir of tolerance alleles [51,55,56,57]. Thus, the new function of resistance genes against various stresses is attracting significant interest. This information should guide the breeding aimed at enhancing crop yields under stressful environments.

Eighty-six *WRKY* genes have been reported in barley [51,56,58,59,60]. Among these, most genes are involved in the defense to biotic stresses. For example, *WRKY23* in *Fusarium graminearum* can improve its resistance through regulating the secondary metabolites. Some WRKYs were induced by abiotic stresses [52,53,55], among which the expression of *HvWRKY1* can significantly improve the barley’s drought tolerance [51,57]. Since the information of the barley genome has been continuously updated and re-annotated, which has an impact on the number of genes identified, the gene length, the number of exons, and the different motifs, we reidentified and characterized the *WRKY* genes with the updated barley genome.

Our previous study identified cold-tolerant and cold-sensitive barley landraces. The transcriptomic analyses of both tolerant and sensitive landraces were performed. Along with the genome data of Ensembl Plants (Morex_v3), we could identify *WRKY* genes from barley landraces. Gene structure, *cis*-acting elements, gene duplication events, chromosome distribution, and comparative phylogenetic analysis were explored. The transcriptomic analyses were conducted to identify the gene expressional model of specific *WRKY* genes under cold stress conditions within two barley landraces. Quantitative real-time PCR (qRT-PCR) was used to verify the expressional levels of *WRKY* genes with different expression models. This study aims to provide new insights into the application of *WRKY* genes of barley landraces. And certain members of WRKYs may be utilized to enhance the quality of barley landraces and serve as resources for the breeding resistance of barley.

## 2. Results

### 2.1. Identification of WRKY Genes

According to the latest genome data of *H. vulgare*, 96 potential WRKYs were uncovered by using HMMER online website, version 3.4 with default parameters. Then, SMART was performed to confirm the existence of the conserved *WRKY* domain. Thus, 96 WRKYs from barley landraces (named HvLWRKYs) were ultimately identified (Table S1).

The physicochemical properties of the *WRKY* proteins, including sequence length, molecular weight (MW), isoelectric point (pI) and other indexes, were determined (Table S1). Among the 96 HvLWRKY proteins, the protein size varied dramatically, with *HvLWRKY59* (136aa) being the smallest protein and *HvLWRKY31* (736aa) being the largest protein. The MW of 96 HvLWRKY proteins ranged from 14.73 kDa to 77.96 kDa with a mean value of 36.96 kDa. The theoretical pI was from 5.10 (*HvLWRKY88*) to 10.17 (*HvLWRKY66*), and nearly half of the WRKY proteins (about 43%) possessed isoelectric points that greater than 7. These suggest that different HvLWRKY proteins might function in various environments.

The average of the instability index of the proteins was 52.93, that is, greater than 40. And the grand average of hydropathicity (GRAVY) was negative and less than 0 for all WRKY proteins (Table S1). The Aliphatic index had a maximum value of 82.75 (*HvLWRKY21*) and a minimum value of 35.96 (*HvLWRKY59*) with a mean of 59.36. Three proteins (*HvLWRKY11*, *HvLWRKY14*, *HvLWRKY35*) with an instability index of less than 40 were regarded as stable proteins, and the remaining were defined as unstable proteins, suggesting that HvLWRKY proteins may be unstable and hydrophilic.

### 2.2. Phylogenetic Relationship Among WRKY Genes

**Figure 1:**
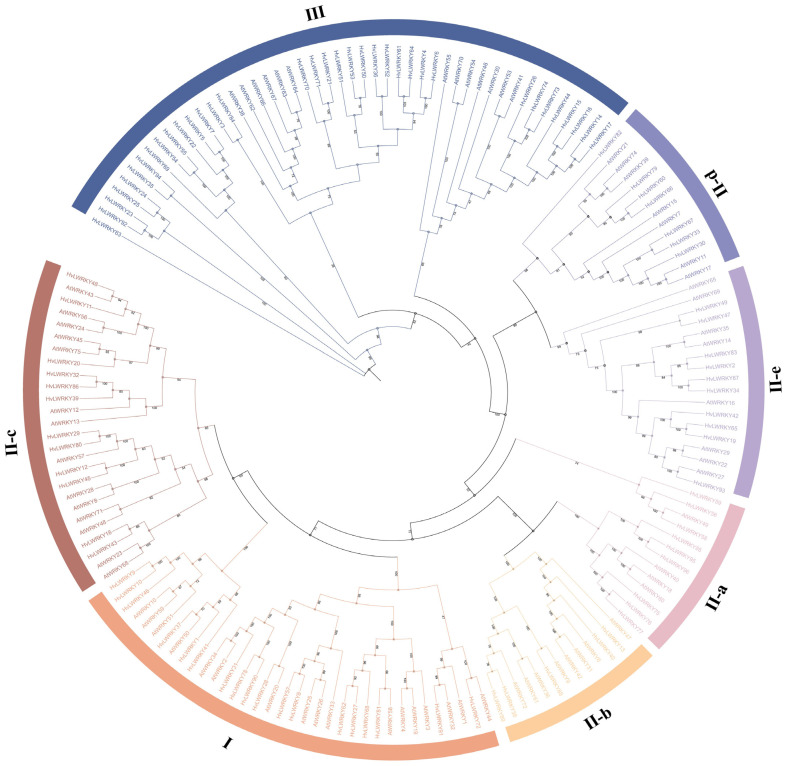
Phylogenetic tree constructed with sequences of the HvLWRKYs and AtWRKYs. Different colors represent different groups.

The identified 96 *WRKY* genes of barley landraces together with the 75 *WRKY* genes from *A. thaliana* were used for phylogenetic analysis, and an unrooted maximum-likelihood phylogenetic tree was generated (Figure 1). The sequences were gathered into three groups (I, II and III). In Group I, 18 HvLWRKYs contained two C_2_H_2_-type WRKY domains. A total of 43 HvLWRKYs were assigned to Group II with a single C_2_H_2_-type domain. The remaining 35 WRKYs were gathered into Group III, which contains the C_2_HC-type WRKY domain. The WRKYs within Group II were further classified into five subgroups—II-a, II-b, II-c, II-d and II-e—according to the conserved sequences of amino acid other than in WRKY domains.

**Figure 2:**
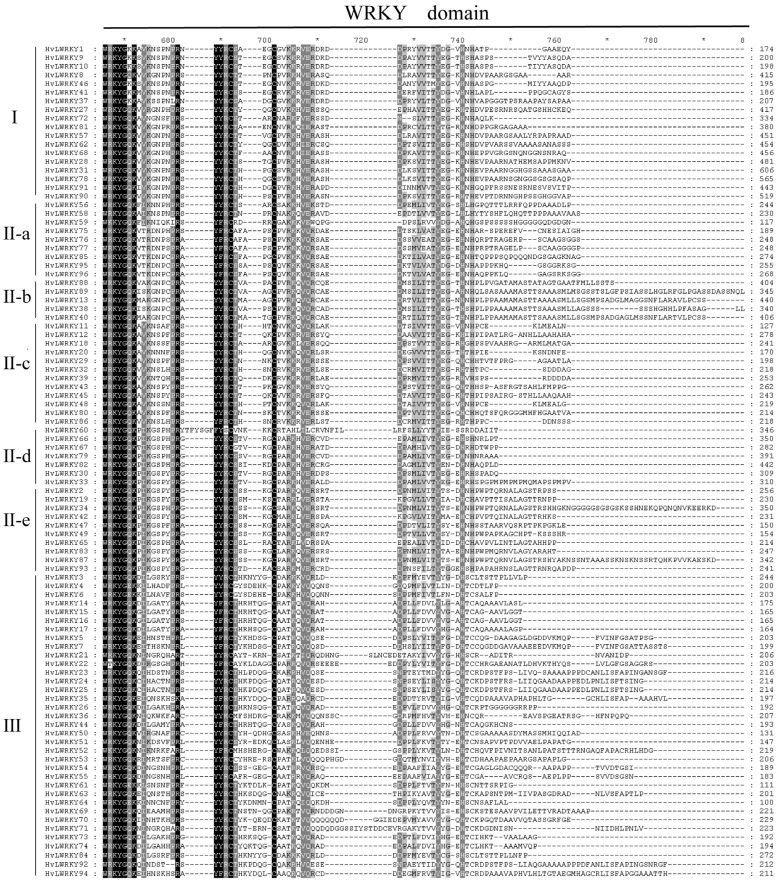
Multiple sequence alignment of the HvLWRKY domains. Shading represents the degree of amino acid identity at each position and black shading indicates 100% sequence identity. ‘*’ indicates positions which have a single, fully conserved residue.

To explore the evolution relationship among *HvLWRKY* genes, we selected all WRKY domains for multiple sequence alignment (Figure 2). A highly conserved WRKY motif was present in the HvLWRKYs. Meanwhile, various other WRKY domains were also observed, including WRKYGEK, WRKYGKK, WKKYGQK, and WTKYGQK, which were mainly found in Groups I, II-a, and III. For example, in Group I, the domains of *HvLWRKY1*, *HvLWRKY9*, *HvLWRKY10*, *HvLWRKY37*, *HvLWRKY41* and *HvLWRKY46* were mutated to WRKYGKK, while the domain of the others was WTKYGQK. The R in the domain of *HvLWRKY22* was mutated to T, forming the WTKYGQK heptapeptide structural domain. The R in the domains of *HvLWRKY52* and *HvLWRKY59* mutated to K, forming the WKKYGQK heptapeptide structural domain. The Q in the domains of *HvLWRKY4*, *HvLWRKY6*, *HvLWRKY61* and *HvLWRKY64* mutated to E, forming the WRKYGEK heptapeptide structural domain. Although WRKYGQK heptapeptide sequences were conserved among HvLWRKYs, low sequence similarity was found outside the structural domain.

### 2.3. Gene Structure and Motif Composition of HvLWRKYs

**Figure 3:**
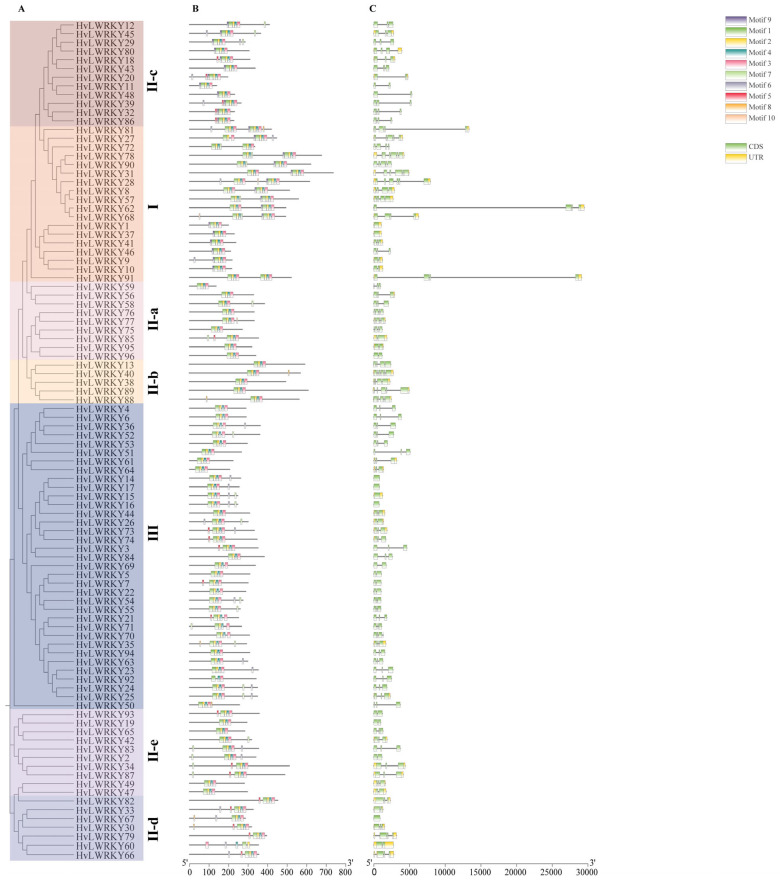
Phylogenetic relationships, conserved motifs, and gene structural analyses of the HvLWRKYs. (**A**) Neighbor-joining phylogenetic tree of the HvLWRKYs. (**B**) Conserved motifs in the HvLWRKYs proteins. The 10 predicted motifs are represented by different colored boxes. (**C**) Gene structure of the HvLWRKYs, and the black line represents an intron.

The organization of each *WRKY* gene exon–intron was analyzed to acquire more insight into the WRKY evolution in barley landraces (Figure 3).

It was found that most *HvLWRKY* genes contained two to four introns. In Group III, the intron numbers varied from zero to three, the most with two introns. The introns in Group I varied from zero to five. A majority of the members in Group II generally contained two introns; however, II-a and II-b members presented more introns compared with the genes in other subclusters of Group II (Figure 3).

To better comprehend the HvLWRKYs’ conservation and diversification, the putative motifs of all HvLWRKY proteins were predicted with MEME motif analysis. A total of 10 motifs were identified (Figure 3B). Among these, motif 1 and 2 were present in all HvLWRKY proteins, which showed that these two motifs were highly conserved. Group I members harbored motifs 1–10. Most of Group III mainly had motif 1–4, while 21 members had motif 5, 6, 7 and 8, respectively. Motif 9 mainly was observed in Group I. As expected, the HvLWRKYs in the same group shared highly similar motif compositions.

The secondary structures of HvLWRKY proteins were predicted using TBtools software, version 2.315. The analysis revealed that these secondary structures primarily consisted of 33.96% alpha helix (Hh), 15.21% extended strand (Ee), and 50.83% random coil (Cc) (Figure S1). No other secondary structures were identified in HvLWRKY proteins. The percentage of Hh varied from 4.23% in *HvLWRKY28* to 32.38% in *HvLWRKY84*. The Ee percentage ranged from 3.69% in *HvLWRKY87* to 13.10% in *HvLWRKY37*. The Cc percentage was from 59.87% in *HvLWRKY95* to 91.35% in HvLWRKY51 (Table S2).

### 2.4. Synteny and Duplication Events in HvLWRKYs

**Figure 4:**
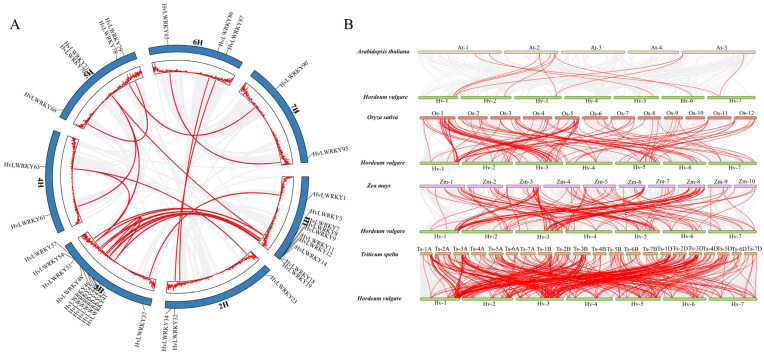
Synteny relationships of *HvLWRKY* genes. (**A**) Duplication events among *HvLWRKY* genes. Gray lines represent all collinear segments and red lines represent duplicated gene pairs. (**B**) Synteny analyses of *WRKY* genes between barley and four representative species. Gray lines represent the collinear regions within barley and other species, and red lines indicate the syntenic gene pairs.

Gene duplication is a primary driver of evolution, providing the raw genetic material for natural selection [61], and also leads to the gene families’ expansion. A total of 25 segmental duplication events were identified in 37 *HvLWRKY* genes (Figure 4A). An enrichment of HvLWRKYs in duplication clusters was found on chromosome 1H and 3H, which included 10 *HvLWRKY* genes. In contrast, tandem duplication events did not occur in *HvLWRKY* genes. These indicated that some *HvLWRKY* genes may have been generated through segmental duplication events, and the evolution of *HvLWRKY* genes could have been, at least in part, driven by such events.

The Ka/Ks ratio measures the selection pressure of the sequences and reflects the species’ evolution selection [29]. All segmental duplicated *HvLWRKY* gene pairs had Ka/Ks < 1 (Table 1), which indicated that these genes experienced strong purifying selective pressure during evolution.

The comparative syntenic maps of *HvLWRKY* family associated with four representative species, including two dicots (*Arabidopsis* and *Zea mays*) and two monocots (*Triticum spelta* and *Oryza sativa*), are presented in Figure 4B. There were 11, 118, 137 and 356 *HvLWRKY* genes that showed a syntenic relationship with *A. thaliana*, *O. sativa*, *Z. mays* and *T. spelta,* respectively (Table S3). More than 75% of the *HvLWRKY* genes showed a syntenic relationship between two Triticeae species, barley and *T. spelta*.

Most *HvLWRKY* genes were connected with two syntenic gene pairs, and others were jointed with three collinear gene pairs (between barley and *T. spelta*/*Z. mays*). Collinear 8, 11, 32, 57, 80, 86 and 90 *WRKY* gene pairs were observed between two barley landraces and all the other four species, showing that these orthologs may be originated before the divergence of dicotyledonous and monocotyledonous plant species.

### 2.5. Cis-Elements in HvLWRKY Promoter Regions

**Figure 5:**
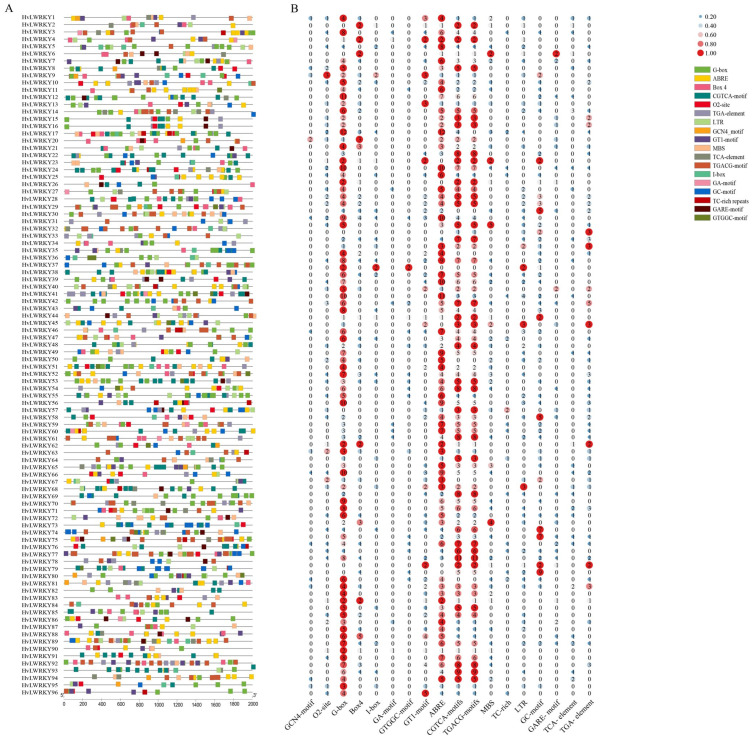
*Cis*-elements in the promoters of *HvLWRKY* genes. (**A**) Different colors of the box indicate different *cis*-acting elements. (**B**) Different numbers represent the number of *cis*-acting elements in each *HvLWRKY* gene.

All *HvLWRKY* genes had several *cis*-acting elements within the regions of their promoter (Figure 5). Finally, 18 representative *cis*-elements were selected for quantitative and functional analysis (Table S4). According to the functions, the *cis*-elements were divided into three categories.

Two categories were related to growth and development, including GCN4-motif and protein metabolism regulative element O2-site. Among these, one was the light-responsive elements (G-box, Box4, I-box, GA-motif, GTGGC-motif, and GT1-motif) [2]. And the other was associated with environmental stress, including ABRE (abscisic acid-responsiveness) [9], CGTCA-motif (MeJA-responsiveness), TGACG-motif (MeJA-responsiveness) [22], MBS (drought stress-responsive element), TC-rich (defense and stress-responsive element), LTR (low-temperature stress responsive element) [37], and GC-motif (enhancer-like element) [24,32]. Among the environmental stress-associated elements in the *HvLWRKY* genes, about half of the genes included ABRE (89 genes involved), TC-rich repeats (16 genes involved), LTR (51 genes involved) and MBS (47 genes involved) [11,13,36]. The third was related to hormone response, such as GARE-motif, TCA-element, and TGA-element.

In summary, the *HvLWRKY* genes in different groups appear to have their own sets of *cis*-responsive elements, suggesting that these groups may have evolved to respond to specific stimuli.

### 2.6. Interaction Network Among HvLWRKY Proteins

**Figure 6:**
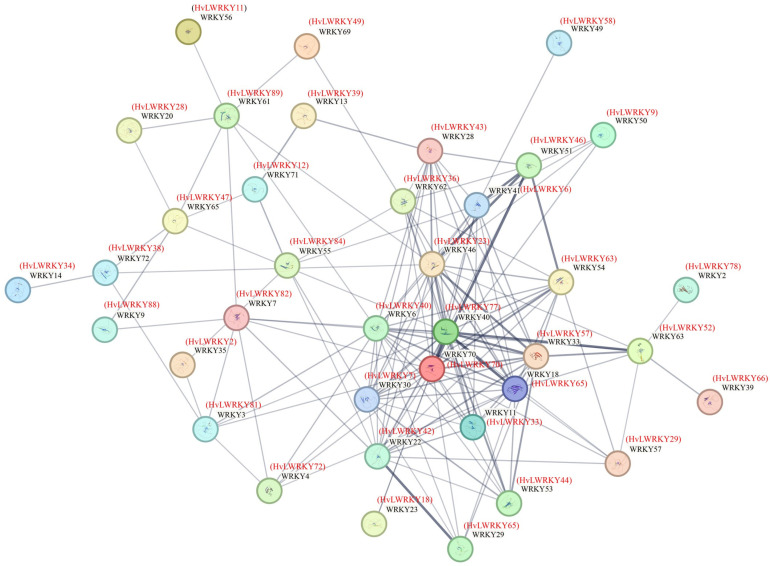
Predicted protein–protein interactions of HvLWRKYs. *Arabidopsis* proteins were taken as a reference for protein–protein interaction network analysis. The thickness of the two connecting lines represents the correlation between the two proteins.

The protein–protein interaction network showed that 38 of the 96 HvLWRKYs exhibited co-expression correlations (Figure 6). Among these, *HvLWRKY77* appeared as a central hub, interacting with *HvLWRKY7*, *70*, *40*, *23*, *36*, *43*, *6*, *63*, *57*, *65*, *44*, *33*, *29*, *18* and *42*, and with other genes over a long distance.

### 2.7. Transcriptome Pattern of HvLWRKY Genes

**Figure 7:**
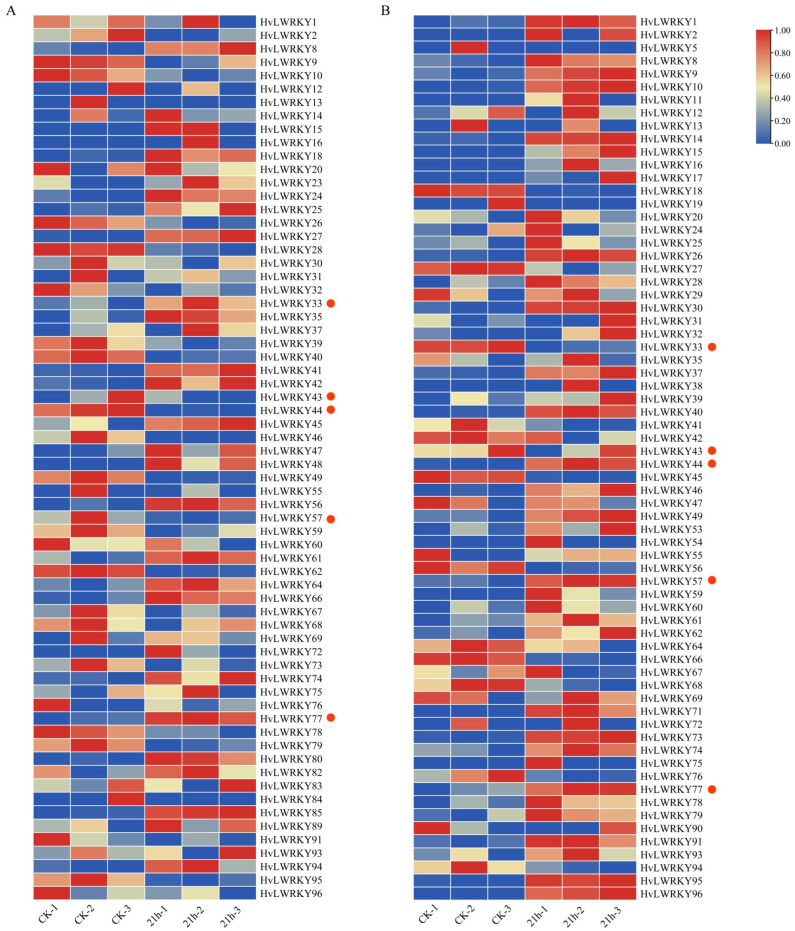
Expression profiles of *HvLWRKY* genes in barley landraces under cold stress. (**A**) Cold-sensitive landrace. (**B**) Cold-tolerant landrace. Red blocks indicate high expression levels, and blue blocks indicate low expression levels. CK denotes the control group, and 21 h represents the duration of cold stress treatment. Red dots represent the genes that primarily interact with *Arabidopsis thaliana*.

To discover the transcriptional mode of *WRKY* genes in two barley landraces, the transcriptional profiles were further analyzed by our lab RNA-seq datasets. Among the *WRKY* genes, 66 HvLWRKYs (Figure 7A, Table S5) and 68 HvLWRKYs (Figure 7B, Table S6) were detected with transcripts in cold-sensitive and cold-tolerant barley landraces, respectively.

Some *HvLWRKY* genes presented the similarity on the transcriptional pattern between cold-tolerant and cold-sensitive barley landraces, for example, *HvLWRKY8* and *HvLWRKY12*. Some had an opposite transcriptional pattern between the two landraces, such as *HvLWRKY66* and *HvLWRKY95*. *HvLWRKY9*, *HvLWRKY41* and *HvLWRKY46* were segmentally duplicated genes, and *HvLWRKY9* and *HvLWRKY46* were detected with a much higher transcriptional level in cold-tolerant than that in cold-sensitive landraces, while *HvLWRKY41* had an inverse transcriptional pattern between two barley landraces.

In addition, eight *WRKY* genes were specifically expressed in each landrace: *HvLWRKY5*, *11*, *17*, *19*, *29*, *38*, *53*, *54*, *71* and *90* in cold-tolerant landrace, while *HvLWRKY23*, *48*, *80*, *82*, *83*, *84*, *85* and *89* in cold-sensitive landrace.

### 2.8. qRT-PCR Validation

**Figure 8:**
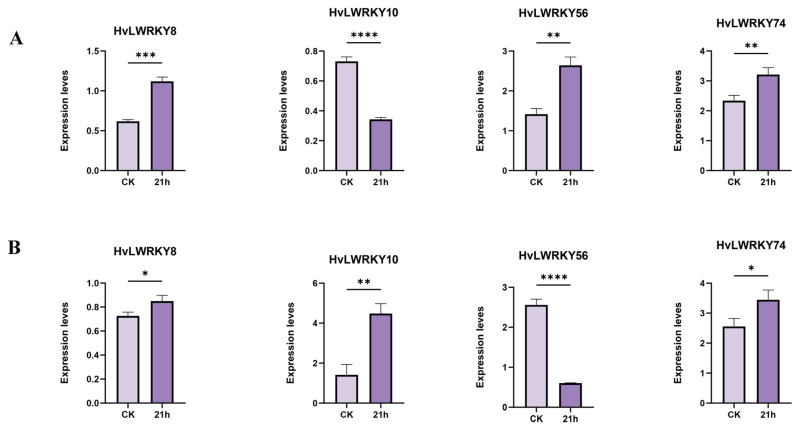
Expression patterns of *HvLWRKY* genes analyzed by qRT-PCR. (**A**) Different genes from cold-sensitive landrace. (**B**) Different genes from cold-tolerant landrace. Vertical bars indicate the mean ± SD value. Statistical comparisons are conducted using one-way analysis of variance (ANOVA) for each variable (**** *p* < 0.0001, *** *p* < 0.001, ** *p* < 0.01, * *p* < 0.05).

Among the four random selected genes (Figure 8A,B), *HvLWRKY8* and *HvLWRKY74* were upregulated in both cold-tolerant and cold-sensitive landraces under cold stress. *HvLWRKY10* was downregulated in cold-tolerant landrace and upregulated in cold-sensitive one, whereas *HvLWRKY56* exhibited the opposite pattern. The qRT-PCT results were consistent with the above expression profiles of *HvLWRKY* genes (Figure 7).

## 3. Discussion

### 3.1. Gene Structure of HvLWRKY Genes

Previous studies identified 86 *WRKY* genes in barley using an early version of *H. vulgare* genome [62]. In the current study, utilizing the latest *H. vulgare* genome data, we discovered 96 WRKY genes, representing an increase of 10 genes compared to the previous findings [62]. Furthermore, our analysis examined the *HvLWRKY* gene expression across different barley landraces under cold stress conditions.

The number of *WRKY* gene varies significantly among different plant species [63,64,65,66,67]. For instance, *Ralstonia solanaceatrum* contains 117 *WRKY* genes [68], *Cyclocarya paliurus* has 88 [69], *Paeonia suffruticosa* has 66 [70], *Amaranthus hypocondriacus* has 55 [66], and *Acer fabri* has 46 [71]. As sequencing technologies advance, more *WRKY* genes will be identified across various species, and the quantification of *WRKY* genes within a single species will become more accurate.

The phylogenetic analysis together with *A. thaliana* [17,32,72] revealed that 96 HvLWRKYs were gathered into Groups I, II (II-a, II-b, II-c, II-d, and II-e) and III (Figure 1). According to Rinerson et al. [23], four major WRKY lineages should be detected in the flowering plants: Groups I + II-c, Groups II-a + II-b, Groups II-d + II-e, and Group III. In the present study, some members of Group I were involved in the same clade as Group II-c (Figure 3). The members of Group II-e were divided into two subclades, which gathered with the members of Group II-d (Figure 3). Thus, our results supported the viewpoints of Rinerson et al. [23].

Meanwhile, Group II-a was considered as the least evolved among all clades of *WRKY* gene family and appeared to be derived from Group II-b [73,74]. Among nine HvLWRKYs of Group II-a, six members gathered with Group II-b (Figure 3). Group III was the most advanced, dynamic, and adaptable gene group, and the number of WRKY III genes may, to some extent, imply the total number of genes in the family [8,36,75]. Our Group III had 35 *HvLWRKY* genes, more than the number in pineapple or *Taraxacum*, and less than in rice or maize [41,75].

Tandem and segmental duplications have both contributed to the expansion of the *WRKY* genes [31,61]. Due to their significant contributions to diverse physiological processes, the WRKYs in plants probably expanded rapidly during the evolution process. Comparison of the *HvLWRKY* genes in the barley genome with the other four species genome found 25 segmental duplication events with 37 *WRKY* genes, while no tandem duplication events occurred in barley landraces (Figure 4). Within *Zea mays* [76], 52 segmental duplication events were found in 78 genes, and there was also no tandem duplication event. However, 5 tandem duplication events and 13 segmental duplications were observed in barley by Zheng et al. [62]. Among 37 duplication *HvLWRKY* genes in this study, 9 were from Group I, 13 from Group III, and 15 from Group II (Figure 3 and Figure 4). This suggested that Group II, especially Group II-c (8 genes involved), may play a major role in the expansion of *HvLWRKY* family, and segmental duplication has been a significant factor in the expansion of *WRKY* genes during the evolution of barley. Further, more than 83% *HvLWRKY* genes showed orthologous relationships with the *WRKY* genes in *T. spelta*, suggesting that the segmental duplication of *WRKY* genes likely occurred in the ancestors of barley and *T. spelta* before their divergence.

The duplication events result in an increase in the number of members within the evolutionary clades, reflecting the process of ongoing evolution [45]. There are other evolutionary processes, for example, the introns gain or loss [45]. An intron embodies a complex structure, such as having donor and acceptor splice sites or being a branch point, and it can arise by segmental duplication [20,77]. *HvLWRKY* genes contain 0–5 introns (Figure 3), suggesting that HvLWRKYs exhibited a low gene structure diversity. Meanwhile, the loss of introns was more likely than the gain of introns [27,78]. If some introns were lost, the length of the genes would be reduced [27]. Group III, II-a, c, d and II-e had only two introns compared with Group I and Group II-b (Figure 3). These five clades evolved from Group I or Group II-b, which were considered as the early lineages. During the evolution, Group I and Group II-b evolved from a common ancestor, and then divergence occurred with motifs 4 and 5 disappearing in Group II-b (Figure 3). This was funded by the fact that II-a evolved from II-b, which was also characterized with a reduction in the length and number of introns. Furthermore, the lowest of introns was presented in Group III, suggesting that *HvLWRKY* genes from Group III might be in a posterior position in evolution (Figure 3).

The loss of domain is a natural phenomenon, reflecting the divergent power of the gene expansion [31]. WRKY transcription factors are characterized by a specific gene structure, which contains a DNA-binding domain featuring a conserved WRKYGQK motif at the N-terminal and a zinc finger motif at the C-terminal [27]. The WRKY motif is highly conserved across many plants featuring the WRKYGQK domain; however, numerous variants within the WRKY domain have been reported in previous studies [3,19]. The presence of WRKYGQK motif variants increase the WRKY protein diversity binding to target genes [19], which was further supported by the different proportion of the secondary structures among HvLWRKY proteins (Table S2 and Figure S1).

The motifs 1 and 2 of Group I were found in other clades (Figure 2), indicating their evolution from a shared origin. Particularly, the collinearity analysis showed that 16 *HvLWRKY* genes had no orthologous genes between barley and *T. spelta*, among which 2 *HvLWRKY* genes belonged to Group I, 3 *HvLWRKY* genes to Group II, and 10 *HvLWRKY* genes belonged to Group III (Figure 4B, Table S3). HvLWRKY6 and 10 proteins containing a mutated WRKY domain of WRKYGEK and WRKYGKK belonged to Group III and II, respectively (Figure 4B). Thus, our data supports that the Group I was the oldest, and Groups II and III evolved from Group I [51,73].

The *cis*-elements in promoter regions act as crucial molecular switches that regulate a broad range of gene networks [75]. *Cis*-acting elements are closely linked to the roles of genes in stress response [51]. WRKYs interact with *cis*-elements downstream of gene promoters to regulate the target gene expression, triggering a series of responses that enhance plant stress resistance [32,33,79]. In this study, a total of 18 *cis*-elements were identified (Figure 5, Table S4). The HvLWRKYs contained some hormone-responsive elements, for example, ABRE, CGTCA-motif, TCA- element, and TGA- element, indicating that these *HvLWRKY* genes are involved in a wide range of hormone-mediated signaling pathways. Importantly, stress-related elements were present in *HvLWRKY* promoter domains, including MBS, TC-rich repeats and LTR. LTR was a *cis*-acting element for low temperature response [75]. Most *HvLWRKY* promoters, such as *HvWRKY1*, *14*, *30* and *68*, had LTR element, which could respond to low-temperature stress (Figure 5).

### 3.2. Expression Pattern of HvLWRKYs Under Cold Stress

WRKYs are involved in responses to abiotic stresses, including low temperature, high temperature, salt, and drought [2,45]. In *Arabidopsis* [8,33] and rice [41,45], at least 26 and 54 *WRKY* genes respond to abiotic stress, respectively. Many WRKYs have an important role in cold stress tolerance in various species [25]. For example, the *CsWRKY21* gene in tea trees is induced by low temperatures and expressed six times more than the control group [80]. In pineapple, seven AcWRKYs were expressed under cold stress [81]. The expression of *PgWRKY26* gene in *Platycodon grandiflorus* significantly increases under cold stress for 6h [82]. A *PmWRKY57* of *Platycodon mume* overexpression line increased the cold tolerance [40].

Low temperatures are a significant environmental stress that impacts barley growth and development, even reducing the yield [11,25]. The expression pattern of all 96 *HvLWRKY* genes was analyzed under cold stress. According to the cold stress expression pattern, more than 50 *HvLWRKY* genes’ expression was significantly induced (Figure 7, Tables S5 and S6). Furthermore, the *WRKY* genes of all groups were involved in response to cold stress in the two barley landraces (Figure 7). In *Liriodendron chinense*, the *WRKY* genes of Group I and II respond to cold stress; however, Group III genes do not respond [50]. We found that some *HvLWRKY* genes (such as *HvLWRKY9*, *10*, *28*, *46*, *57*, *78*) of Group I were upregulated in two landraces, indicating Group I may be the main cold-stress-regulatory genes in two landraces.

*HvLWRKY* genes were differentially expressed between cold-tolerant and cold-sensitive landraces (Figure 7). Under cold stress, some genes, including *HvLWRKY8* and *HvLWRKY77,* were upregulated in both the landraces (Figure 7 and Figure 8). In the interaction network (Figure 6), *HvLWRKY77* was found to be homologous to *AtWRKY40* in *Arabidopsis* and acted as a central nodes. *AtWRKY40* is involved in both biotic and abiotic stress responses in plants [83]. Transgenic lines expressing *AtWRKY40* exhibit enhanced drought and osmotic tolerance. Additionally, *AtWRKY18*, *AtWRKY40*, and *AtWRKY40* form a highly interactive regulatory network that influences gene expression related to plant defense and stress responses. *HvLWRKY65* showed high sequence similarity with *AtWRKY18*, suggesting a potential interaction with *HvLWRKY77*.

More *HvLWRKY* genes were upregulated in cold-tolerant or upregulated in cold-sensitive landrace (Figure 7A). qRT-PCR analysis of the randomly selected genes revealed that *HvLWRKY10* exhibited high expression levels in the cold-tolerant landrace and low expression in the cold-sensitive landrace. In contract, *HvLWRKY39* and *HvLWRKY56* were downregulated in the cold-tolerant landrace but upregulated in the cold-sensitive one (Figure 7 and Figure 8). *HvLWRKY33*, *43*, *44*, and *57*, being homologous with *AtWRKY11*, *28*, *53*, and *33*, respectively (Figure 6), showed a similar expression pattern (Figure 7). The overexpression of *AtWRKY11* and *AtWRKY28* enhances osmotic tolerance in *A. thaliana* [63,64]. Similarly, the overexpression of *AtWRKY33* increases tolerance to salt, heat, and drought. *AtWRKY53* directly represses CDK-related kinase 5 (CRK5), thereby increasing osmotic tolerance. Both *AtWRKY53* and *AtWRKY33* participate in the salicylic acid (SA) and jasmonic acid (JA) pathways to regulate disease resistance [63,64]. Consequently, we speculated that *HvLWRKY33*, *43*, *44*, and *57* may regulate osmotic tolerance in two barley landraces, helping them resist cold stress and enhance pathogenic tolerance through SA, JA pathways (Figure 5). In addition, *HvLWRKY1*, *53*, *71*, *74* and *96* genes were specifically expressed in the cold-tolerant landrace, and *HvLWRKY 32*, *39*, *59* and *67* specifically expressed in the cold-sensitive landrace. These genes are important regulators, which may play roles in response to cold stress.

The divergence in gene expression significantly contributes to the preservation of duplicated genes [19]. Some paralogous gene pairs showed different responses to cold stress, indicating that they played various roles in barley landraces for responding to cold stress [29]. For example, *HvLWRKY9* was highly expressed in the cold-sensitive type under cold stress, while its paralogue gene *HvLWRKY41* was downregulated. In the cold-tolerant landrace, these two genes presented opposite expression patterns: *HvLWRKY9* was downregulated, and *HvLWRKY41* was upregulated (Figure 7A). Some *HvLWRKY* genes and their paralogues, such as *HvLWRKY18* and *HvLWRKY45*, exhibited similar profiles of high transcript abundance in the cold-tolerant or cold-sensitive landraces under cold stress—they were upregulated in cold-tolerant landraces but downregulated in cold-sensitive ones (Figure 7B)—suggesting that they may have redundant functions.

## 4. Materials and Methods

### 4.1. Identification and Characterization of WRKY Genes

The genomic sequences of cultivated barley variety ‘Morex_v3′ were obtained from Ensembl Plants (https://plants.ensembl.org/index.html, accessed on 4 August 2024) [84]. First, the published WRKY protein sequences of *Arabidopsis thaliana* were used as queries, BlastP was carried out to identify WRKY sequences against the coding sequences of barley genomes in TBtools software, version 2.315, (https://github.com/CJ-Chen/TBtools, accessed on 4 August 2024) [85]. Second, the coding sequences were detected using the PlantTFDB (v4.0, Plant Transcription Factors Database) [73] to validate WRKY sequences. Then, the Hidden Markov Model (HMM) profiles of WRKY DNA binding domain (PF03106) were performed for searching barley proteome sequences via the HMMER program with an E-value cutoff of 0.0001. The above sequences of WRKY protein were confirmed with SMART databases (http://smart.embl-heidelberg.de/, accessed on 4 August 2024) [86]. Ninety-six *WRKY* family genes were finally obtained, and the identified genes were named HvLWRKYs accordingly.

Subsequently, the online software ExPASy, version 3.0, (https://web.expasy.org/protparam/, accessed on 6 August 2024) [74] was utilized to analyze the protein properties, including the length of the protein, molecular weight (MW), theoretical isoelectric point (pI), grand average of hydropathicity (GRAVY), aliphatic index (AI), and instability index (II).

### 4.2. Phylogenetic Tree Construction and Multiple Sequence Alignment

The domains of *HvLWRKY* genes were aligned using ClustalW program of MEGA software, version 7.0) [87] with default parameters. ProtTest program (v3.4.2) was adopted to estimate the best-fit model of maximum likelihood (ML) trees. The ML trees were constructed by IQ-tree program, version 2.4.0 [88]. The consistency of ML trees was validated with an ultrafast bootstrap value of 1000. The phylogenetic tree was visualized using the iTOL online website (http://itol.embl.de/, accessed on 8 August 2024). Then, the deduced amino acid sequences within WRKY domains were adjusted by GeneDoc software, version 2.7 [89].

### 4.3. Gene Structure, Motif Composition of HvLWRKYs

All *WRKY* gene structures were predicted with Gene Structure Display Server (GSDS), version 2.0, (http://gsds.cbi.pku.edu.cn/, accessed on 10 August 2024) [90]. The exon–intron structure of each *WRKY* gene was identified using the coding sequence (CDS) description and genome annotation. Motif analysis was performed using the MEME online tool in classic mode, version 5.5.5, (https://meme-suite.org, accessed on 10 August 2024) [91] with the parameters as follows: number of repetitions, any; maximum number of motifs, 10; and optimum width of each motif, between 6 and 300 residues.

The *cis*-acting elements in *WRKY* genes promoter sequences (upstream 2000bp) were detected through the PlantCare website (http://bioinformatics.psb.ugent.be/webtools/plantcare/html/, accessed on 11 August 2024) [92]. The secondary structure of the WRKY proteins was predicted with TBtools software, version 2.315, (https://github.com/CJ-Chen/TBtools, accessed on 11 August 2024) [85].

### 4.4. Synteny and Duplication Events Among HvLWRKY Genes

The gene duplication events were analyzed using Multiple Collinearity Scan toolkit (MCScanX, version 1.0) with default parameters [93]. The syntenic relationships of *WRKY* genes were constructed using MCScanX software, version 1.0 [93]. Meanwhile, the non-synonymous (Ka) to synonymous (Ks) ratios per site between gene pairs were calculated by the Ka/Ks Calculator in TBtools, version 2.315 [85,94].

### 4.5. Protein–Protein Interaction Among HvLWRKY Proteins

The HvLWRKY sequences were uploaded to STRING database (https://cn.string-db.org, accessed on 15 August 2024) [95], and the WRKY sequences of *A. thaliana* served as a reference for analyzing protein–protein interactions.

### 4.6. Transcriptome Analysis of HvLWRKY Genes

In the preliminary experiment involving 120 barley landraces provided by the Institute of Wheat Research of Shanxi Agricultural University, two barley landraces were selected: one cold-tolerant and one cold-sensitive. After seed germination at room temperature, six seedlings of similar growth from each landrace were placed into pots. Two groups were set up: one for low-temperature treatment and another for normal-temperature culture as a control. At the three-leaf stage, the low-temperature treatment group was exposed to −6 °C for 21 h, as determined by the preliminary experiment.

After treatments, the same portions of leaves were flash-frozen with liquid nitrogen and sent to Lc-Bio Technologies Co., Ltd. (Hangzhou, China) for transcriptome sequencing. Using the transcriptome datasets, the expression patterns of HvLWRKYs were analyzed. Each sample set was used for three biological replicates. The RPKM value was used to represent gene expression, with log10-transformation applied. Then the expression of *HvLWRKY* genes was displayed on a heatmap with the TBtools software [61,82], and the expression levels were exhibited through a color bar that transitioned from blue to red.

Finally, the quantitative real-time PCR (qRT-PCR) analysis was conducted using a LightCycler Fast Real-Time PCR system from Roche, Switzerland. Primers were designed with PRIMER 5.0 software and are listed in the additional file Table S2 (Tables S2 and S7). *HvLWRKY8*, *10*, *56* and *76* representing various expression patterns were randomly selected, and *HORVU.MOREX.r3.4HG0396310* derived from barley was used as the internal reference gene for normalization. The reaction was performed as follows: 95 °C for 3 min, 95 °C for 10 s, 68 °C for 15 s and 40 cycles. Each reaction had three biological and technical replicates, and the gene expression levels were calculated using 2^−ΔΔCT^ method with t values [66]. Microsoft Excel 2010 was utilized for data entry and statistical analyses, while GraphPad Software (v7.0, San Diego, CA, USA) was employed to create bar graphs. Error bars in expression graphs indicate the mean ± standard deviation (SD) of replicates.

## 5. Conclusions

In the present study, the conserved motifs, evolutionary relationships, and gene structures of the 96 identified HvLWRKYs were examined. The expressional models of *HvLWRKY* genes in two barley landraces under cold stress were explored. *HvLWRKY* genes may play important roles in barley growth and development. Some genes were found differentially expressed between the two barley landraces, indicating different responses to cold stress for different landraces. Particularly importantly, *HvLWRKY33*, *43*, *44*, *57*, *65*, and *77* (Figure 6, Figure 7 and Figure 8) are homologous with the relative *AtWRKY* genes in *A. thaliana*. They are suggested to regulate the abiotic and pathogen resistance of two barley landraces via SA and JA pathways [63,64]. However, more evidence is needed to further understand the function of these genes. Future studies should concentrate on utilizing modern tools, including single-cell technologies, multi-omics integration, and AI-driven prediction, to elucidate the specific roles of HvLWRKYs in barley landraces. Thus, understanding the full extent of the regulatory functions and the dynamic regulatory network of *HvLWRKY* genes involved would provide a solid foundation for exploring the tolerance mechanism and identifying new genes resistant to cold stress for barley breeding.

## Figures and Tables

**Table 1:** Duplicated *HvLWRKY* genes in barley landraces.

Duplicate Gene Pair	Ka	Ks	Ka/Ks
*HvLWRKY5*/*HvLWRKY51*	0.750	1.198	0.626
*HvLWRKY7*/*HvLWRKY54*	0.548	0.921	0.595
*HvLWRKY9*/*HvLWRKY46*	0.333	1.096	0.304
*HvLWRKY11*/*HvLWRKY48*	0.152	0.544	0.280
*HvLWRKY9*/*HvLWRKY41*	0.628	1.216	0.516
*HvLWRKY18*/*HvLWRKY45*	0.522	0.909	0.574
*HvLWRKY8*/*HvLWRKY57*	0.263	0.449	0.585
*HvLWRKY12*/*HvLWRKY45*	0.265	0.518	0.511
*HvLWRKY14*/*HvLWRKY44*	0.504	0.741	0.679
*HvLWRKY18*/*HvLWRKY43*	0.475	0.944	0.503
*HvLWRKY19*/*HvLWRKY42*	0.334	0.762	0.438
*HvLWRKY1*/*HvLWRKY37*	0.424	1.018	0.416
*HvLWRKY5*/*HvLWRKY70*	0.695	1.173	0.592
*HvLWRKY23*/*HvLWRKY63*	0.495	1.053	0.470
*HvLWRKY32*/*HvLWRKY86*	0.210	0.597	0.351
*HvLWRKY34*/*HvLWRKY87*	0.278	0.907	0.307
*HvLWRKY41*/*HvLWRKY46*	0.470	1.950	0.241
*HvLWRKY43*/*HvLWRKY45*	0.423	0.714	0.593
*HvLWRKY51*/*HvLWRKY63*	0.806	1.496	0.539
*HvLWRKY51*/*HvLWRKY70*	0.693	1.903	0.364
*HvLWRKY54*/*HvLWRKY71*	0.778	1.589	0.490
*HvLWRKY61*/*HvLWRKY70*	0.786	1.438	0.546
*HvLWRKY66*/*HvLWRKY79*	0.377	1.725	0.219
*HvLWRKY78*/*HvLWRKY90*	0.338	1.222	0.276
*HvLWRKY85*/*HvLWRKY95*	0.288	0.617	0.466

## Data Availability

The datasets used and/or analyzed during the current study are available from the corresponding author on reasonable request.

## Supplementary Materials

The following supporting information can be downloaded at: https://www.mdpi.com/article/10.3390/ijms26146948/s1.
