# Supplementary material for: Characteristics and Expression Profiles of Identified WRKY Genes in Barley Landraces Under Cold Stress
Source: Int J Mol Sci. 2025 Jul 19;26(14):6948. doi: 10.3390/ijms26146948 (PMC12296175; doi:10.3390/ijms26146948)
Supplement: Supplementary file 1 [file ijms-26-06948-s001.zip › Figure S1.pdf]

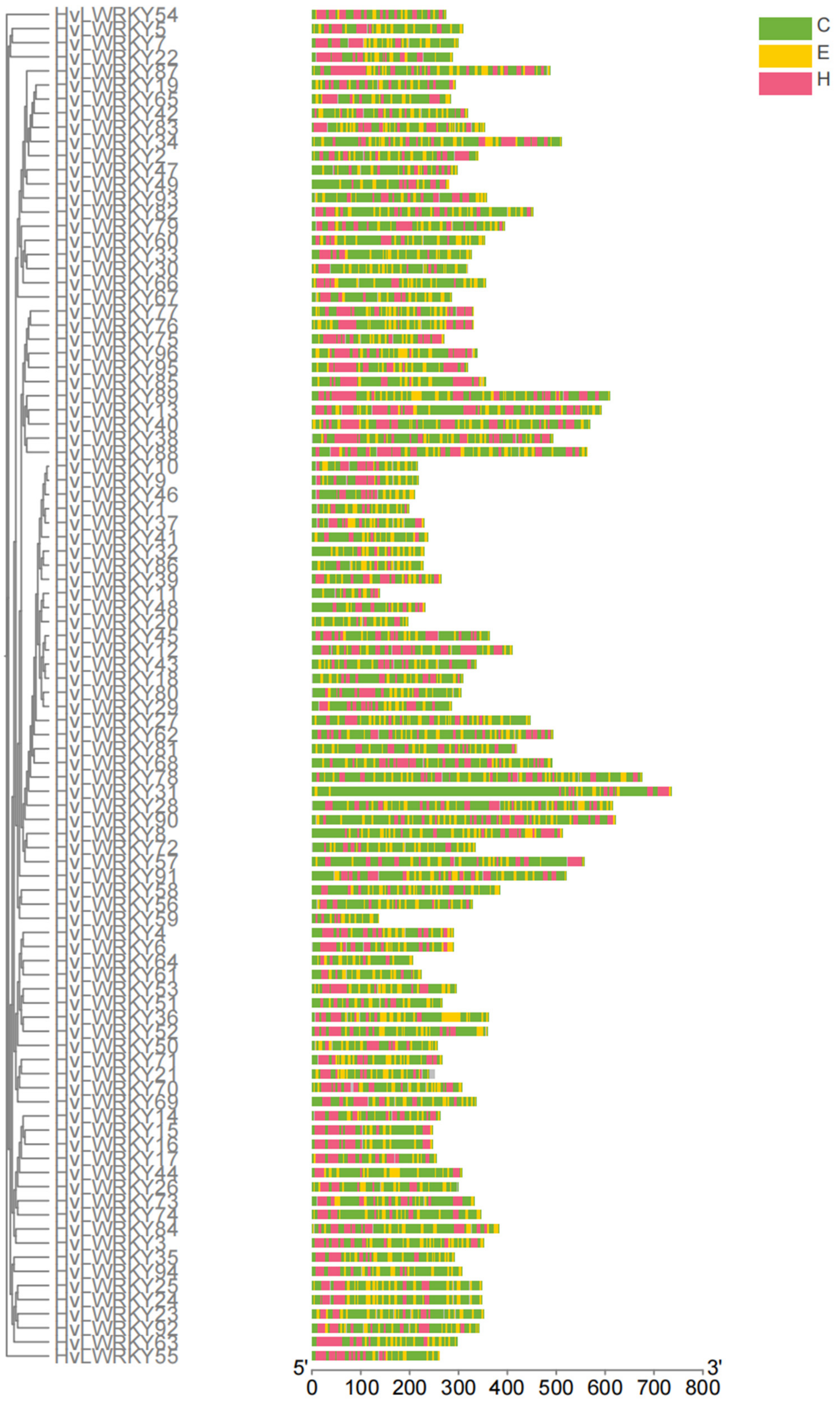

Figure S1. The secondary structure of HvLWRKY proteins. C represents random coil, E represents extended strand, H represents alpha helix
